# Porous Scaffolds Based on Polydopamine/Chondroitin Sulfate/Polyvinyl Alcohol Composite Hydrogels

**DOI:** 10.3390/polym15020271

**Published:** 2023-01-05

**Authors:** Zuwu Tang, Meiqiong Yu, Ajoy Kanti Mondal, Xinxing Lin

**Affiliations:** 1 School of Materials and Environmental Engineering, Fujian Polytechnic Normal University, No.1, Campus New Village, Longjiang Street, Fuzhou 350300, China; 2 College of Material Engineering, Fujian Agriculture and Forestry University, Fuzhou 350108, China; 3 Leather Research Institute, Bangladesh Council of Scientific and Industrial Research, Dhaka 1350, Bangladesh

**Keywords:** composite hydrogel, cytocompatibility, porosity, porous scaffolds

## Abstract

In this paper, porous scaffolds based on composite hydrogels were fabricated using polydopamine (PDA), chondroitin sulfate (CS), and polyvinyl alcohol (PVA) via the freezing/thawing method. Different characteristics of the prepared composite hydrogels, including the pore sizes, compression strength, lap shear strength, mass loss, and cytocompatibility were investigated. Scanning electron microscope images (SEM) displayed the hydrogel pore sizes, ranging from 20 to 100 μm. The composite hydrogel exhibited excellent porosity of 95.1%, compression strength of 5.2 MPa, lap shear strength of 21 kPa on porcine skin, and mass loss of 16.0%. In addition, the composite hydrogel possessed good relative cell activity of 97%. The PDA/CS/PVA hydrogel is cytocompatible as a starting point, and it can be further investigated in tissue engineering.

## 1. Introduction

Tissue engineering is an interdisciplinary field that applies the principles of engineering and the life sciences toward the development of biological substitutes that restore, maintain, or improve tissue function [1]. Artificial materials are emerging as appealing materials for tissue engineering applications. At present, artificial materials are used in tissue engineering, including polymer materials, such as hydrogels [2,3,4,5,6,7], metal materials [8,9], and inorganic nonmetallic materials [10,11].The metal materials and inorganic nonmetallic materials are greatly limited because of poor biocompatibility, biodegradability, and poor interface binding with surrounding tissues, and polymer materials are widely used to construct scaffold materials due to good biocompatibility, biodegradability, and adjustable structure [12]. However, some polymer materials are restricted due to poor mechanical properties, concerns about potential immunogenic reactions and infection [13]. Therefore, it is still a great challenge to construct a kind of scaffold material with strong interfacial force, mechanical strength, and degradation matching with surrounding tissues.

Polyvinyl alcohol (PVA) is a water-soluble, biodegradable, biocompatible, nontoxic, and hydrophilic polymer [14], which has potential applications for biomedicine, bioelectronics, and bioengineering [15,16,17,18]. However, pure PVA-based hydrogels have poor mechanical properties, which restrict their practical applications [19]. Therefore, other alternatives are required to improve its mechanical strength, such as blending [20], post-treatment [21], and cross-linking [22], which further improve the applications of hydrogels.

Chondroitin sulfate (CS) is a type of sulfated glycosaminoglycan, which is widely distributed on the ECM and cell surface of animal tissues [23]. CS can improve bone regeneration by controlling its signal transduction pathway or by increasing the efficacy of arrangement of the growth factor [24] and plays an important role in regulating cell functions [25]. CS-based hydrogels exhibit good biological activity and wound healing ability, which can help them restore joint function affected by arthritic activity. However, native CS-based hydrogels have low mechanical properties and weak cell affinity [23], which are limited in clinical studies. Therefore, it is of great significance in tissue engineering to develop a novel CS-based hydrogel.

Dopamine (DA) is secreted from marine mussels. It is easy to polymerize polydopamine (PDA) nanoparticles through oxidation cross-linking reactions in aqueous solution [26], which has potential applications for biomedicine, bioelectronics, and coatings [27,28,29]. PDA has been proven to enhance cell attachment and proliferation [30] as well as have strong adhesion to various substrates [31]. Because PDA has reactive groups, such as amino groups and catechol groups [32]. PDA-based hydrogels are prepared by physical cross-linking of catechol groups. However, the low conjugation of catechol groups results in insufficient mechanical properties [33], and therefore the hydrogels are greatly limited in their application.

In this work, we designed a PDA/CS/PVA composite hydrogels with porous structures, tissue adhesiveness, and cytocompatibility through the freezing/thawing method, which was formed by physically cross-linking via the reversible hydrogen bonds among the functional hydroxyl, carboxyl, and amide groups. The mechanical properties, rheological behavior, swelling behaviors, mass loss, and cytocompatibility of the composite hydrogel were studied. On the basis of its facile preparation, porous structure, outstanding mechanical strength, and cytocompatibility, the composite hydrogel is a starting point and it can be further investigated in tissue engineering.

## 2. Experiments

### 2.1. Materials

Polyvinyl alcohol (PVA-1799, 110,000–130,000 g/mol and a 98–99% hydrolyzation degree), 3,4-dihydroxyphenylalanine hydrochloride (DOPA·HCl), chondroitin sulfate (CS), ammonium hydroxide (NH_3_·H_2_O, 28%~30%), and ethyl alcohol were purchased from Aladdin Biochemical Technology Co., Ltd., Shanghai, China. A concentration of 1.5 times simulated body fluid (SBF, sterility, AR) was purchased from Beijing InnoChem Science & Technology Co., Ltd., Beijing, China, and NIH 3T3 cells were obtained from Shanghai Fuxiang Biological Technology Co., Ltd., Shanghai, China. 3-(4,5-Dimethylthiazol-2-yl)-2,5-diphenyltetrazolium bromide (MTT) was supplied by Sigma. Dulbecco’s modified eagle medium (DMEM) was obtained from Hyclone. Fetal bovine serum (FBS) was purchased from Gibco. Penicillin/streptomycin was obtained from Beyotime. All reagents were analytical grade and used without further purification. Ultrapure water (18.2 MΩ·cm) obtained by a water purifier (Sichuan Water Purifier Co., Ltd., Chengdu, China) was used for all experiments.

### 2.2. Preparation of Polydopamine (PDA)

Briefly, 4.0 mL of NH_3_·H_2_O was added to 80 mL of ethyl alcohol and 180 mL of ultrapure water, which could form a uniform mixture with stirring under 30 °C for 30 min. Then, 1.0 g of DOPA·HCl was dissolved in 20 mL of ultrapure water and added to the above mixture solution. The reaction was maintained under 30 °C for 24 h [34]. After dialyzing for 3days, PDA was obtained by freeze-drying.

### 2.3. Preparation of PDA/CS/PVA Composite Hydrogels

The PDA/CS/PVA composite hydrogels with various PDA contents were prepared as follows. Firstly, PVA was dissolved in ultrapure water to form a 10 wt% PVA solution by stirring vigorously at 90 °C. Secondly, PDA and CS were added to the PVA solution, which was stirred and sonicated for 30 min to form a uniform mixture. Thirdly, for the freezing/thawing process: the hydrogels were first cooled to −18 °C for 24 h and then thawed at room temperature over 3 h. This process was repeated three times to prepare the PDA/CS/PVA composite hydrogels [35]. The pure PVA and CS/PVA hydrogels were prepared by the same procedure. The detailed composition of PDA/CS/PVA composite hydrogels is shown in Table 1.

### 2.4. Characterization

The surface morphology of the hydrogels was observed using a scanning electron microscopy (JEOL, JSM-5600 V, Tokyo, Japan) with 10 kV acceleration voltages. The samples were sprayed by galvanic platinum deposition for enhancement of conductivity before measurements.

The dynamic rheological behavior of the hydrogels was characterized with a stress-controlled rheometer, Rotational Rheometer MARS III Haake (MARS III, Verden, Germany), at room temperature. During strain scanning tests, the samples deformed under different shear strains. The storage modulus (G′) and loss modulus (G″) were recorded by frequency sweeps at angular velocities ranging from 0.1 to 100 Hz.

The hydrogels have been investigated by X-ray photoelectron spectroscopy (XPS, Thermo Scientific ESCA-LAB 250Xi, Waltham, MA, USA) with a standard Al *K*α X-ray source, which measured wide scan spectra and high-resolution scans. The X-ray beam was operated at a current of 10 mA and an acceleration voltage of 15 kV.

The size of PDA particles was determined from a Marvin laser granulometer (Zetasizer Nano ZS90, Worcestershire, UK).

### 2.5. Mechanical Properties

The compression test of the hydrogels was carried out using a universal mechanical testing machine (KJ-1065B, Kejian Instrument Co., Ltd., Dongguan, China). The hydrogels with diameter of 25 mmand height of 12 mm were prepared for the compression test. The original area of the samples was recorded (*S*), and the compressive load was recorded (*F*) when the compressive strain reached 80%. Three duplicate samples of each hydrogel were employed to test the compression stress. The compression stress (*σ*) of the hydrogels was calculated using the following formula [36]:

*σ* = *F*/*S* (1)

The lap shear strength was measured on porcine skin using the same testing machine. Porcine skin was cleaned with deionized water before testing. The hydrogels were coated on the substrates. Then, two substrates were overlapped (the area of overlap is called *A*, mm^2^) and cured for 48 h at room temperature. Finally, the overlapped area of adhesive joint was pulled off and the maximum loading force was recorded (*P*, N). The hydrogels were tested in shear with single lap joint porcine skin following the ASTM D1002 standard [37].Three duplicate samples of each hydrogel were employed to test the lap shear strength. The lap shear strength (*τ*, MPa) was tested and calculated as follows [38]:

*τ* = *P*/*A* (2)

### 2.6. Swelling Ratio

The swelling experiment was performed to evaluate the swelling behavior of the hydrogels in ultrapure water. The weight of the dry sample was recorded (*W*_0_) and immersed in 50 mL of ultrapure water at room temperature. The weight of the swollen sample was recorded (*W*_t_) after 24 h. Five duplicate samples of each hydrogel were employed to test the swelling ratio. The swelling ratio was determined by the following equation [39]:

Swelling ratio (%) * = * (*W*_t_*− W*_0_)*/W*_0_ × 100% (3)

### 2.7. Porosity

The porosity of the hydrogels was measured [40]. The dry sample was weighed (*m*_1_). The pycnometer was filled with ultrapure water (*ρ*_w_) and weighed (*m*_2_). The sample was put into the pycnometer, which was immersed with ultrapure water and then filled with water and weighed (*m*_3_). The sample filled with water was taken out and the remaining water and the pycnometer was weighed (*m*_4_). The total volume (*V*_P_) of the porous hydrogel, the total volume (*V*_S_) of the solid hydrogel, as well as the porosity (*ε*) were calculated by the following equations [12]:

*V*_P_ = (*m*_3_− *m*_4_− *m*_1_)/*ρ*_w_ (4)

*V*_s_ = (*m*_1_+*m*_4_− *m*_3_)/*ρ*_w_ (5)

*ε = V*_P_/(*V*_P_+*V*_s_) × 100% (6)

### 2.8. Mass Loss

The mass loss of composite samples was assessed in simulated body fluid (SBF) under pH = 7.4. The samples were washed and cleaned using ultrapure water with ultra-sound, dried in avacuum oven, and ultraviolet-sterilized for 30 min. The sample weights were recorded (*W*_0_) separately and the samples were stored in an incubator at 37 °C with 100 rpm/min shaking speed. The samples were taken every week and SBF was changed. The samples were washed with ultra-pure water and dried for 24 h before their dry weight was weighed (*W*_t_). Three duplicate samples of each hydrogel were employed to test the mass loss. The mass loss was calculated by the following equation [41]:

Mass loss = (*W*_0_− *W*_t_)/*W*_0_ × 100% (7)

### 2.9. Cytocompatibility

NIH 3T3 cells were cultured in DMEM medium containing 10% FBS and 10 units/mL penicillin/streptomycin at 37 °C and 5% CO_2_ in a saturated humidity incubator. The samples were cut into cuboids (2 mm × 1 mm × 1 mm). Then, the samples were washed with PBS solution and ultraviolet-sterilized for 30 min. After sterilization, the samples were placed into 96-well plates with a density of 10^5^ cells/well. After 1day and 5 days of culture, the NIH/3T3 cells proliferated, and the viability of the NIH/3T3 cells on the samples was evaluated using an MTT assay. The MTT assay was performed by following these steps: addition of 20 μL of MTT, incubation at 37°C for 4 h, suction of the supernatant after centrifugation, dissolution of formazan using DMSO. The optical density (OD) values of the control group and the hydrogels group at 490 nm were measured using a microplate reader (Infinite F50, TECAN, California, USA) [42]. Four duplicates from each group were used to assess the cytocompatibility of hydrogels. The relative cell viability was calculated by the following equation [43]:

Relative cell viability = *A*_adhesive_/*A*_control_ × 100% (8)

where *A*_adhesive_ is the absorbance for cells cultured in the adhesive piece and *A*_control_ is the absorbance for cells cultured in cell culture medium.

### 2.10. Statistical Analysis

All the quantitative results are expressed as mean ± standard deviation (SD). Statistical analysis was carried out by means of one-way analysis of variance (ANOVA). A *p*-value less than or equal to 0.05 was considered statistically significant.

## 3. Results

### 3.1. Design Concept and Characterization of PDA/CS/PVA Composite Hydrogels

**Figure 1:**
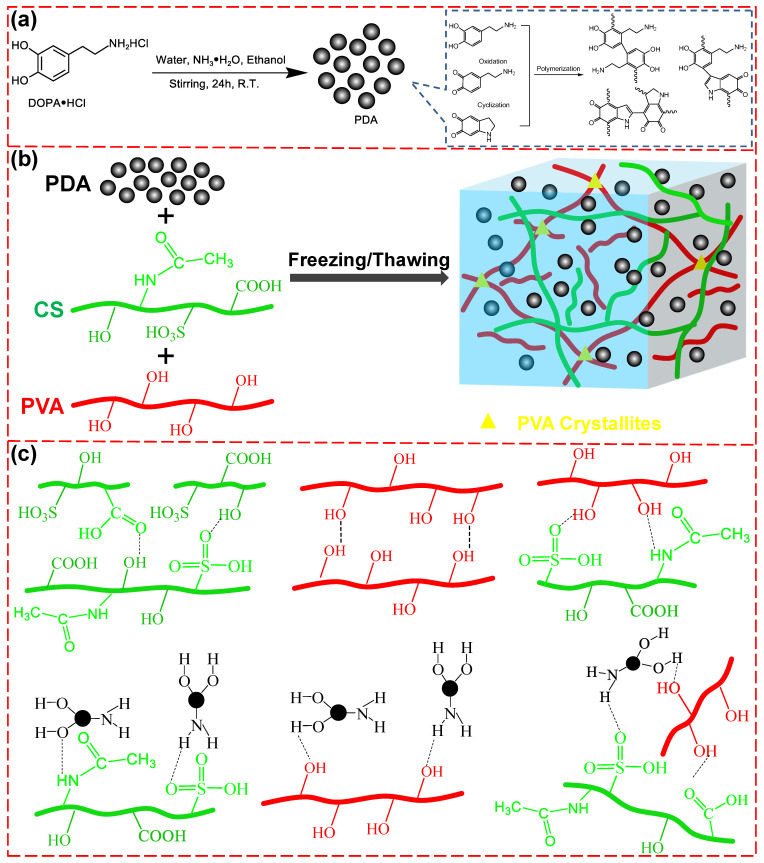
(**a**) Preparation scheme of PDA. (**b**) Schematic illustration for the formation of PDA/CS/PVA composite hydrogel. (**c**) The possible mechanism of PDA/CS/PVA composite hydrogel.

PDA/CS/PVA composite hydrogels were prepared via the following steps: Firstly, dopamine hydrochloride was self-polymerized into PDA (Figure 1a). Secondly, PDA and CS were added to PVA solution under vigorous stirring and sonicating to form a uniform mixture. Thirdly, the composite hydrogels were prepared through the freezing/thawing method repeated three times (Figure 1b). The network structure was formed based on the crystallization and entanglement of the PVA chains and the hydrogen bond of PVA and PDA, PVA and CS, and CS and PDA (Figure 1c).

**Figure 2:**
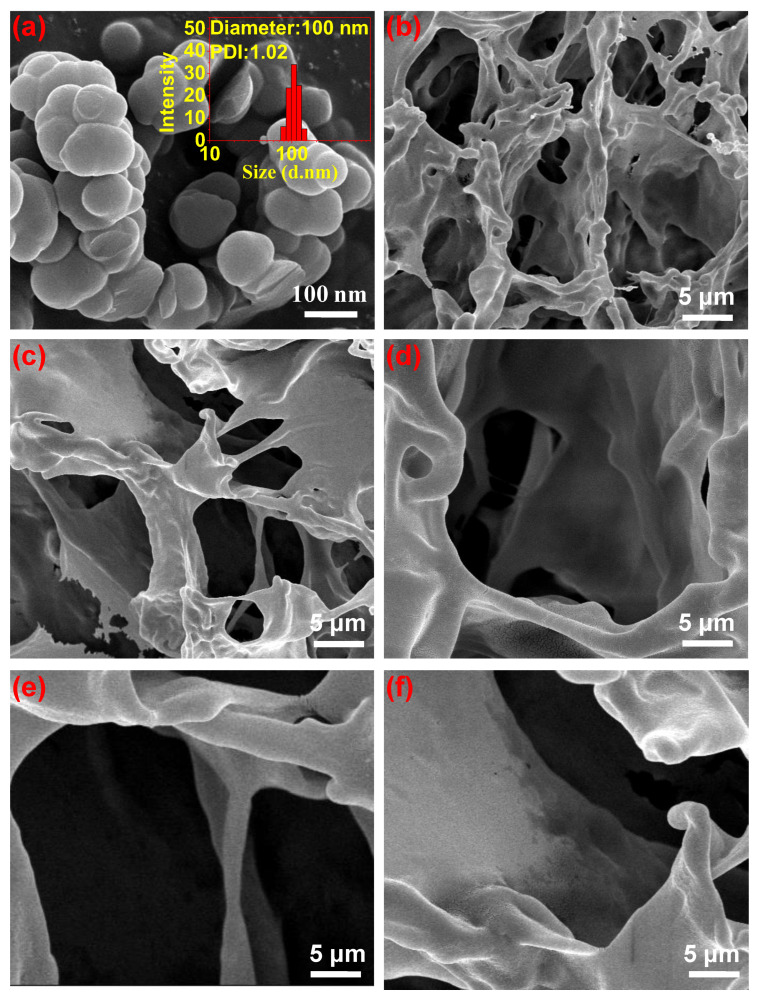
SEM of (**a**) PDA particles, (**b**) PVA hydrogel, (**c**) CS/PVA hydrogel, (**d**) PDA_5_/CS/PVA hydrogel, (**e**) PDA_10_/CS/PVA hydrogel, (**f**) PDA_15_/CS/PVA hydrogel.

The particle size and distribution of PDA were measured by Malvern particle size analyzer, and the morphology of PDA nanoparticles and the hydrogels were observed by SEM, as shown in Figure 2.

The porosity is shown in Table 2. The porosity of pure PVA hydrogel was 59.6%. When CS was added into PVA solution to form CS/PVA hydrogel, the porosity of CS/PVA hydrogel was increased to 79.4%. However, when the PDA content continued to increase, the porosity of the hydrogels gradually increased. When PDA content was 15 mg, the maximum porosity was 95.1%.

**Figure 3:**
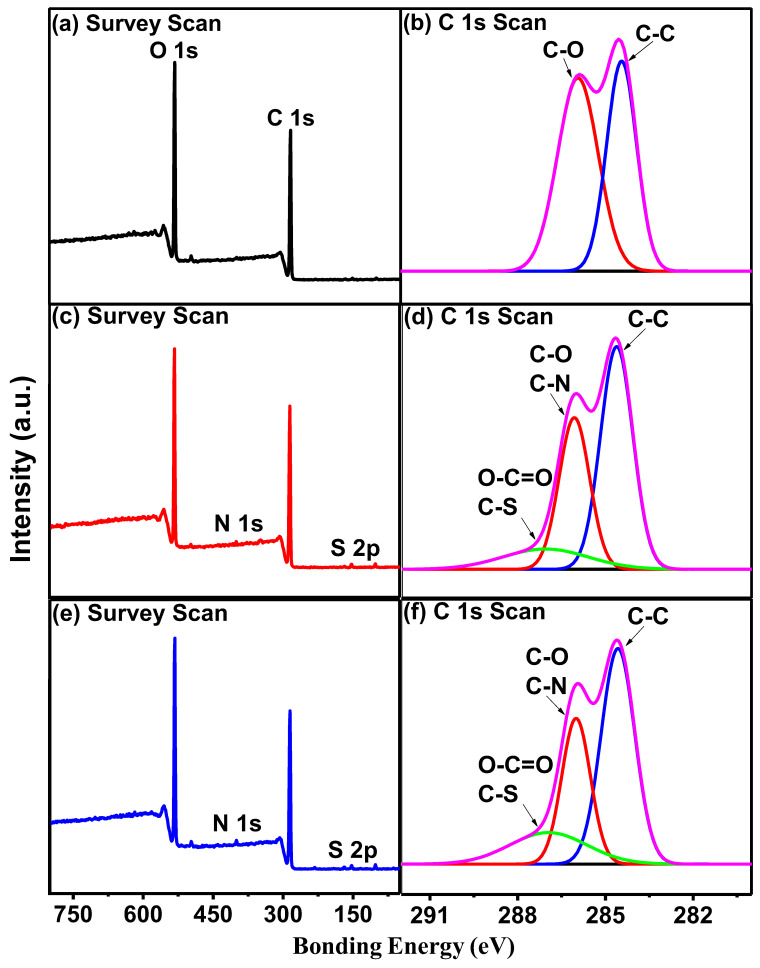
XPS spectra of (**a**,**b**) PVA hydrogel, (**c**,**d**) CS/PVA hydrogel, (**e**,**f**) PDA/CS/PVA hydrogel.

The composition of PVA hydrogel, CS/PVA hydrogel and PDA/CS/PVA hydrogel was analyzed using XPS, as shown in Figure 3, and the position of peaks is listed in Table 3.

**Figure 4:**
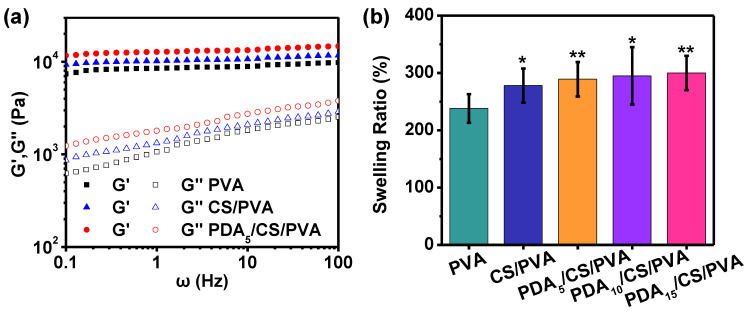
(**a**) Storage modulus (G′) and loss modulus (G″) of the hydrogels. (**b**) Swelling ratio of the hydrogels. (* *p* < 0.05, ** *p* < 0.01).

The storage modulus (G′) and loss modulus (G″) of the hydrogels are shown in Figure 4a. For all hydrogels, G′ value was higher than the G″ value in the whole frequency range.

Figure 4b shows the swelling ratio of the hydrogels. The swelling ratio of PVA hydrogel was 238% (*p* > 0.05). When CS was added to the PVA solution to form CS/PVA hydrogel, the swelling ratio increased to 278% (*p* < 0.05). With the increase in PDA content, their swelling ratio gradually increased. When the PDA content was 15 mg, the maximum swelling ratio reached 300% (*p* < 0.01).

### 3.2. Mechanical Properties

**Figure 5:**
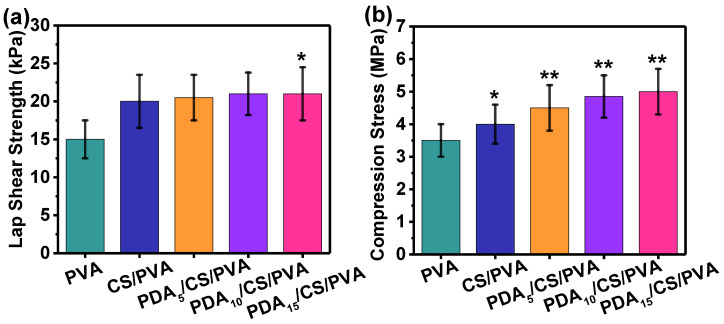
(**a**) Lap shear strength of the hydrogels on porcine skin. (**b**) Compression strength of the hydrogels. (* *p* < 0.05, ** *p* < 0.01).

The lap shear strength of the hydrogels is shown in Figure 5a. The pure PVA hydrogel was only 15 kPa on porcine skin (*p* > 0.05). When CS was added to the PVA solution, the lap shear strength increased to 19 kPa. However, when PDA was added to CS/PVA mixed solution, the lap shear strength of the PDA/CS/PVA composite hydrogel was increased to 21 kPa (*p* < 0.05).

Figure 5b shows the compression strength of the hydrogels. The compression strength of pure PVA hydrogel was 3.6 ± 0.6 MPa. When CS was added to the PVA solution, the compression strength of CS/PVA hydrogel increased to 4.2 ± 0.7 MPa. When PDA was added to CS/PVA mixed solution, the compression strength of the PDA/CS/PVA composite hydrogel gradually increased to a maximum of 5.2 MPa (*p* < 0.01).

### 3.3. Mass Loss

**Figure 6:**
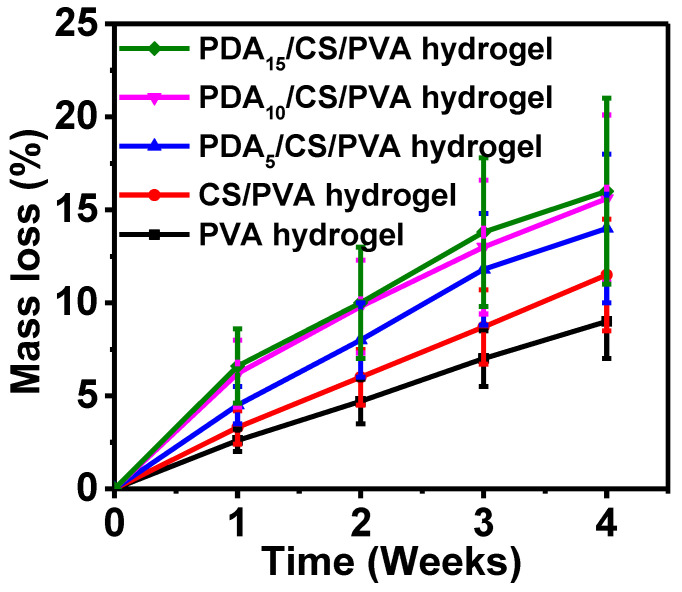
Mass loss of the hydrogels at different times.

The hydrogels were placed in the SBF for the mass loss test. As shown in Figure 6, it can be seen that with the extension of time, the mass loss of the hydrogels increased gradually. The mass loss values of pure PVA hydrogel and PDA_15_/CS/PVA hydrogel were 8.5% and 16.0%, respectively. After 4 weeks, the mass loss of the hydrogels was less than 20.0%.

### 3.4. Cytocompatibility

**Figure 7:**
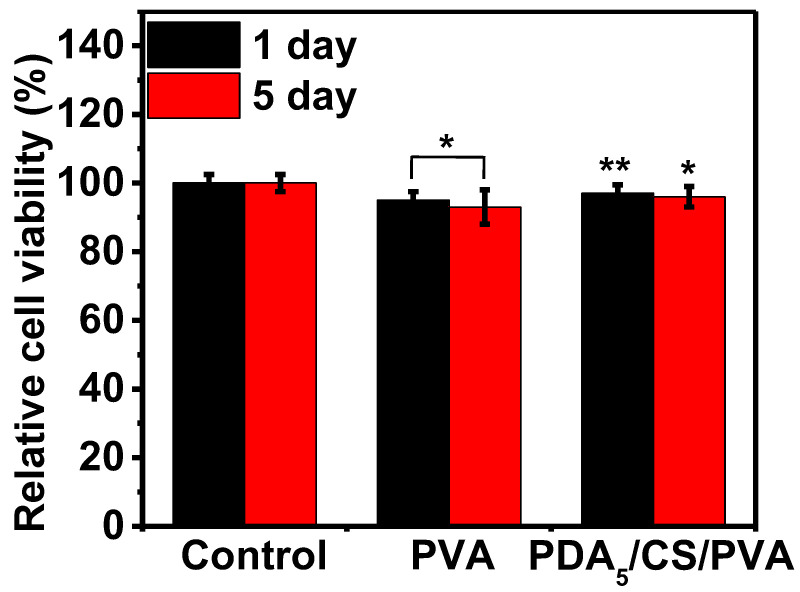
Relative cell viability of the hydrogels. (* *p* < 0.05, ** *p* < 0.01).

NIH 3T3 cells were used to assess the cytocompatibility using the time points of MTT assay at 1 day and 5 days, as shown in Figure 7. For a comparison, a blank group was used as a control. After the first day of cell culture, the relative cell viability of PVA hydrogel was 95% (*p* < 0.05) and that of PDA/CS/PVA hydrogel was 98% (*p* < 0.01). Similarly, after 5 days of cell culture, the relative cell viability of PVA hydrogel was 93% (*p* < 0.05) and that of PDA/CS/PVA hydrogel was 97% (*p* < 0.05).

## 4. Discussion

PDA/CS/PVA composite hydrogels were prepared through the crystallization and entanglement of the PVA chainsand the hydrogen bonds of PVA and PDA, PVA and CS, and CS and PDA. The network of PDA/CS/PVA composite hydrogels could be recombined, fractured, and homogenized by multipoint hydrogen bonds. The crystallization of PVA chains could be easily restored via the freezing/thawing method, which could endow the hydrogel with mechanical properties. PDA and CS were introduced into the PVA hydrogel, which could improve energy dissipation and porosity.

From SEM observation, PDA nanoparticles were uniform spherical particles with a particle size of 100 ± 20 nm (Figure 2a). PVA hydrogel, CS/PVA hydrogel, and PDA/CS/PVA hydrogel were filled with a large number of interconnected irregular pores. With the addition of CS and different PDA content, the pore size of hydrogels increased (Figure 2b–f). PDA/CS/PVA composite hydrogel displayed a three-dimensional porous structure with uneven pore size ranging from 20 μm to 100 μm.

In addition to the pore size of the hydrogels, porosity is also one of the important indicators of scaffold materials [44]. The high porosity can increase the surface area of scaffold materials, which is conducive to the adhesion and growth of a large number of cells, and the substance exchange of cells in the scaffold materials also depends on structures with high porosity. The porosity of pure PVA hydrogel was only 59.6%. When PDA content increased from 0 mg to 15 mg, the porosity increased from 79.4% to 95.1%. Compared to pure PVA and CS/PVA hydrogels, the porosity of PDA/CS/PVA composite hydrogels increased significantly. Compared with the physicochemical properties of other porous scaffolds, although titanium, poly(L-lacticacid), and *β*-Tricalcium phosphate-based scaffolds had large pore sizes, their porosity was low (Table 4). Poly(L-glutamic acid) and polyacrylamide-based scaffolds had high porosity, but the pore sizes were small. The porosity and large pore size of our prepared PDA/CS/PVA composite hydrogels are superior. Therefore, PDA/CS/PVA composite hydrogel could meet the porous requirements of the scaffolds.

In the XPS spectrum, the survey scan of PVA hydrogel had O 1s and C 1s peaks, which were at 532.1 eV and 285.1 eV, respectively [49]. In the spectra of C 1s scan, the C-O and C-C peaks of PVA hydrogel appeared at 286.0 eV and 284.5 eV, respectively [50]. Two new peaks (N 1s and S 2p) at 400.1 eV and 153.1 eV appeared in the spectrum of the CS/PVA hydrogel. The peaks of CS/PVA hydrogel at 287.1 eV, 286.0 eV and 284.5 eV were mainly ascribed to the bonds of O=C-O/C-S, C-N/C-O, and C-C, respectively [51], which indicated CS/PVA hydrogel contains CS. Two new peaks (N 1s, S 2p) at 400.1 eV and 153.1 eV appeared in the spectrum of PDA/CS/PVA hydrogel. The peaks of PDA/CS/PVA hydrogel at 287.1 eV, 286.0 eV, and 284.5 eV were attributed to the bonds of O=C-O/C-S, C-O/C-N, and C-C, respectively [52], which indicated the PDA/CS/PVA hydrogel contained CS and PDA.

The dynamic rheological responses of the PVA hydrogel, CS/PDA hydrogel, and PDA/ CS/PDA hydrogel were reported. In the whole frequency range, the G′ value was higher than the G″ value, indicating a gel network with elastic behavior rather than viscous nature [53,54]. Compared with the G′ value of the three hydrogels, the G′ value of the PDA/CS/PVA hydrogel was the greatest and the G′ value of PVA hydrogel was the smallest in the whole frequency range, indicating that the mechanical properties of the PDA/CS/PVA composite hydrogel with a hierarchical network structure were more robust than that of the CS/PVA and PVA hydrogels [35].

From the swelling ratio analysis, the results showed that the hydrogels exhibited an increased swelling ratio. When PDA and CS were not added, the swelling ratio of PVA hydrogel was 238%. When the PDA content increased from 0 mg to 15 mg, the swelling ratio increased from 278% to 300%. The reason is that a large number of hydrophilic groups (PDA and CS) were introduced into the PDA/CS/PVA composite hydrogel, which could form hydrogen bonds with water and absorb a large amount of water to increase the swelling ratio.

From the mechanical test point of view, it seemed PVA improved the strength in any composition, which could be due to the hydrogen bonds between PDA, CS, and PVA restricting the motion of the matrix while promoting rigidity. When PDA content increased from 0 mg to 15 mg, the lap shear strength was increased from19 kPa to 21 kPa. However, pure PVA hydrogel had 15 kPa. The compression strength was increased from 4.2 MPa to 5.2 MPa. Because CS and PDA were introduced into the PVA network with hydrogen bonds for energy dissipation. Some of the hydrogen bonds were destroyed during the compression test. However, when the pressure disappeared, new hydrogen bonds could be reformed. It was reported that the compression strength of alginate-based porous scaffold was 2.7 MPa [12], and the compression strength of poly(L-lactic acid) microsphere-incorporated calcium alginate/hydroxyapatite porous scaffolds was about 0.6 MPa [46]. The results of the mechanical test indicated that PDA/CS/PVA hydrogels had excellent elasticity and toughness.

The mass loss of the hydrogels was obtained. With the extension of time, the mass loss of the hydrogels increased gradually. The mass loss of the hydrogels with PDA was higher than that of the hydrogels without PDA, and the mass loss of the hydrogels gradually increased with the increase of PDA content. The mass loss of PDA_15_/CS/PVA hydrogel was more than that of all the hydrogels. This is because the PDA_15_/CS/PVA hydrogel had a larger size and a higher swelling ratio, which made the SBF permeate into the inner gel and promoted its dissolution quickly.

The cytocompatibility of PDA/CS/PVA composite hydrogels was tested. After the first day and 5 days of cell culture, the relative cell viability of the PVA hydrogel and PDA/CS/PVA hydrogels was more than 90%. It was reported that relative cell viability below 70% was considered toxic [55]. Therefore, the NIH 3T3 cell test showed that the PDA/CS/PVA composite hydrogel was non-toxic and cytocompatible. It is understandable that PVA, CS, and PDA are biocompatible, leading to cytocompatible PDA/CS/PVA composite hydrogels.

## 5. Conclusions

In this work, porous scaffolds based on the composite hydrogels were fabricated using polydopamine (PDA), chondroitin sulfate (CS), and polyvinyl alcohol (PVA) via the freezing/thawing method. The properties of the composite hydrogels with different PDA contents were measured. The conclusions can be summarized as follows:

(1) PDA/CS/PVA hydrogel appeared in two new peaks (N 1s, S 2p)at 400.1 eV and 153.1 eV in the spectrum. The peaks of the PDA/CS/PVA hydrogel at 287.1 eV, 286.0 eV and 284.5 eV were attributed to the bonds of O=C-O/C-S, C-O/C-N and C-C, respectively. This result indicated the PDA/CS/PVA hydrogel was prepared successfully.
(2) PDA/CS/PVA composite hydrogels had good porosity and mechanical properties. With the increase in PDA content, the porosity and mechanical properties of the hydrogel increased. When the PDA content was 15 mg, the composite hydrogel exhibited excellent porosity of 95.1%, compression strength of 5.2 MPa, and good lap shear strength of 21 kPa on porcine skin. In addition, the mass loss of the hydrogels was less than 20.0%. The composite hydrogel also had good cytocompatibility. PDA/CS/PVA hydrogel is cytocompatible as a starting point and it can be further investigated in tissue engineering.

## Figures and Tables

**Table 1:** Composition of PDA/CS/PVA composite hydrogels.

Samples	PVA (g)	CS (mg)	PDA (mg)	Water (g)
PVA Hydrogel	2	0	0	18
CS/PVA Hydrogel	2	100	0	18
PDA_5_/CS/PVA Hydrogel	2	100	5	18
PDA_10_/CS/PVA Hydrogel	2	100	10	18
PDA_15_/CS/PVA Hydrogel	2	100	15	18

**Table 2:** Porosity of the hydrogels.

Samples	*ε*_1_/%	*ε*_2_/%	*ε*_3_/%	$\bar{\varepsilon }$/%
PVA Hydrogel	58.5	59.7	60.6	59.6
CS/PVA Hydrogel	79.9	77.8	80.7	79.4
PDA_5_/CS/PVA Hydrogel	80.2	81.6	81.0	80.9
PDA_10_/CS/PVA Hydrogel	85.1	83.3	85.7	84.7
PDA_15_/CS/PVA Hydrogel	94.7	95.8	94.9	95.1

**Table 3:** XPS spectral peak of the hydrogels.

Samples	Bonding Energy (eV)	Assignment
PVA hydrogels	O 1s	532.1 eV
	C 1s	285.1 eV
	C-O	286.0 eV
	C-C	284.5 eV
CS/PVA hydrogels	O 1s	532.1 eV
	C 1s	285.1 eV
	N 1s	400.1 eV
	S 2p	153.1 eV
	O=C-O/C-S	287.1 eV
	C-N/C-O	286.0 eV
	C-C	284.5 eV
PDA/CS/PVA hydrogels	O 1s	532.1 eV
	C 1s	285.1 eV
	N 1s	400.1 eV
	S 2p	153.1 eV
	O=C-O/C-S	287.1 eV
	C-O/C-N	286.0 eV
	C-C	284.5 eV

**Table 4:** Physicochemical properties of typical porous scaffolds.

Scaffold	Pore Size (μm)	Porosity	Biocompatiblity	References
Titanium	490	68%, 88%	Bioinert	[9]
*β*-Tricalcium phosphate	350–500	81%	Bioactive	[45]
Poly(L-lacticacid)	>100	90%	Biocompatible	[46]
Polyacrylamide	23–52	98%	Biocompatible	[47]
Poly(L-glutamic acid)	20–50	95%	-	[48]

## Data Availability

Data is contained within the article.
